# EVALUATION OF THE QUALITY AND RELIABILITY OF INFORMATION ON ADHESIVE CAPSULITIS (FROZEN SHOULDER) ON THE SOCIAL MEDIA PLATFORM INSTAGRAM

**DOI:** 10.1590/1413-785220263402e297030

**Published:** 2026-05-11

**Authors:** Douglas Hideo Higuchi, Fabio Teruo Matsunaga, Guilherme Satake, Augusto Yassuo Mathuy, Marcel Jun Sugawara Tamaoki

**Affiliations:** 1 Universidade Federal de Sao Paulo, Departamento de Ortopedia e Traumatologia, Escola Paulista de Medicina, Grupo de Cirurgia do Ombro e Cotovelo, Sao Paulo, SP, Brazil.

**Keywords:** Internet, Social Media, Frozen shoulder, Adhesive capsulitis, Internet, Mídias Sociais, Ombro congelado, Capsulite adesiva

## Abstract

**Objective:**

To assess the quality and reliability of information on adhesive capsulitis provided to the public on Instagram.

**Method:**

The 100 most relevant posts on #adhesivecapsulitis and the 100 posts on #frozenbrown hashtags were analyzed on a single day. The evaluation was carried out by three professionals at different levels - a general practitioner, an orthopedist, and a shoulder and elbow surgeon – using the Global Quality Score (GQS), the modified DISCERN, and the Frozen Shoulder Specific Score (FSSS). In addition, interobserver agreement was checked.

**Results:**

The majority (65.5%) of the 200 posts analyzed presented incomplete information, scoring 1 or 2, representing low scientific foundation and limited clinical applicability. The average scores on the three scores indicated unsatisfactory quality of the content. However, agreement between the evaluators was high, demonstrating the reliability of the methods used.

**Conclusion:**

Although Instagram is widely used as a source of health information, it has poor-quality content on adhesive capsulitis. These findings reinforce the importance of medical guidance and the production of more accurate, evidence-based information materials. **Level of evidence IV; observational, descriptive, cross-sectional**.

## RESUMO

**Objetivo:**

Avaliar a qualidade e a confiabilidade das informações sobre capsulite adesiva fornecidas ao público no Instagram.

**Método:**

Foram analisados, em um único dia, os 100 posts mais relevantes da hashtag #capsuliteadesiva e os 100 posts da #ombrocongelado. A avaliação foi realizada por três profissionais de níveis distintos — médico generalista, ortopedista e cirurgião de ombro e cotovelo – utilizando os escores Global Quality Score (GQS), DISCERN modificado e Frozen Shoulder Specific Score (FSSS). Além disso, a concordância interobservador foi verificada.

**Resultados:**

Os 200 posts analisados apresentaram, em sua maioria (65,5%), informações incompletas, pontuando 1 ou 2, representando baixa fundamentação científica e limitada aplicabilidade clínica. As pontuações médias nos três escores indicaram qualidade insatisfatória dos conteúdos. No entanto, a concordância entre os avaliadores foi alta, demonstrando a confiabilidade dos métodos empregados.

**Conclusão:**

O Instagram, embora amplamente utilizado como fonte de informações em saúde, apresenta conteúdos de baixa qualidade sobre capsulite adesiva. Esses achados reforçam a importância da orientação médica e da produção de materiais informativos mais precisos e baseados em evidências. **Nível de evidência IV; observacional descritivo transversal**.

## INTRODUCTION

Adhesive capsulitis, also known as "frozen shoulder," is a clinically relevant condition affecting about 2-5% of the general population.^1^ The disease causes loss of both passive and active range of motion in the shoulder, primarily affecting patients between 40 and 60 years of age.^2^ The disease significantly affects shoulder function due to pain and restriction of range of motion, particularly in external rotation. The condition is often idiopathic, but has a higher prevalence in individuals with *diabetes mellitus* and hypothyroidism. The natural course of adhesive capsulitis traditionally involves a painful phase followed by a recovery phase, usually lasting one to two years, with an expectation of complete resolution of symptoms. However, untreated adhesive capsulitis can lead to persistent functional limitations, challenging the notion of spontaneous complete recovery.^3^

Your risk factors include diabetes, hyperthyroidism, and previous cervical surgeries. Mostly, the cases are self-limiting^4^, but non-surgical treatments, such as physical therapy, corticosteroid injection, ultrasound-guided hydro-distension, hyaluronic acid injection, or even surgical treatment, such as manipulation under anesthesia or arthroscopic release, may be part of the treatment for some patients.^5^ In this context, obtaining correct and reliable information is necessary for a better understanding of the symptoms, prognoses, and treatment possibilities of the referred condition.

Among social networks, Instagram is a fast and widely used tool today, with Brazil ranked third in terms of users, totaling 134 million.^6^ However, the quality of the information provided by this platform appears to be questionable, as already evidenced by previous studies conducted on other topics, such as prostate cancer^7^, oral cavity cancer^8^, and hypothyroidism^9^.

The hypothesis of the present study is that the content on Instagram about adhesive capsulitis is low in quality and reliability. With the growing number of social media users^2^, the number of *posts* providing information, whether for laypeople or professionals, has been gradually increasing. In this sense, it is necessary to evaluate the reliability of the information provided on Instagram, as this network reaches a large number of people. Therefore, a careful and objective evaluation of this information, often provided to people seeking a better understanding of the disease, is necessary. By knowing the quality and reliability of this information, it will be possible to inform patients and affirm the accuracy of the information provided, as well as anticipate misinformation and questions they may raise.

The quality of information on adhesive capsulitis on digital platforms has already been evaluated by various authors, particularly on YouTube. In general, the results of these studies affirmed that the information about adhesive capsulitis / frozen shoulder is of low quality and limited educational value, suggesting that patients rely on more trustworthy and reliable sources in search of knowledge.^10-12^

It is worth noting that the quality of information on Instagram regarding adhesive capsulitis has not yet been studied. Thus, this research proposed a systematic analysis of the *posts* on Instagram with the *hashtags* #capsuliteadesiva (#adhesivecapsulitis) and #ombrocongelado (#frozenshoulder), using the DISCERN^13^, Global Quality^14^, and *Frozen Shoulder Specific Score* (FSSS).^10^

## OBJECTIVE

To evaluate the quality of the *posts* about adhesive capsulitis and frozen shoulder on the social media platform Instagram, using three different objective scores: modified DISCERN, GQS, and FSSS, in addition to inter-rater reliability.

## METHOD

A scientific, exploratory, and cross-sectional study was conducted, analyzing, in a single day, 200 *posts* most relevant to the hashtags #adhesivecapsulitis (100 *posts*) and #frozenshoulder (100 *posts*), from a newly created Instagram account, configured as Brazilian and belonging to a fictitious female user aged 50 years. Contents that were not in Portuguese, not publicly accessible, or not related to adhesive capsulitis were excluded by two of the evaluators (DH and AM); if there was disagreement, a third evaluator (FM) would decide on inclusion.

The *posts* included were independently evaluated by three different evaluators: an orthopedic resident, a board-certified orthopedic surgeon, and a shoulder and elbow surgeon. The scores used to evaluate the content were the modified DISCERN, GQS, and FSSS.

The modified DISCERN (adapted from DISCERN, a tool created for evaluating written health content) assesses the reliability of the information source provided. The standards, which varied in scores from 0 to 5, followed these criteria: (A) Were the objectives clear and achieved? (A) Is a reliable source of information used? (C) Is the information provided balanced and impartial? (D) Are there other resources listed for patient reference? A score of 0 or 1 indicates a lack of information, a score between 2 and 3 indicates that some information is present, and a score of 5 indicates all available information.

The FSSS is a specific score for adhesive capsulitis or frozen shoulder that also assesses the quality of information and consists of 18 items, derived from guidelines published by the American Academy of Orthopaedic Surgeons, with a score from 0 to 18. This score evaluates information about common patient presentations and symptoms, anatomy, diagnosis and assessment, treatment, and postoperative evolution.

The Global Quality Score (GQS) assesses content quality based on a score from 0 to 5. Scores of 0 and 1 are for those with low-quality information, missing information, or little utility for patients; 5 points for content with excellent-quality information that is relevant and useful for the patient.

After checking the parametricity of the data using the Shapiro-Wilk test (p > 0.05), the scores were quantified and evaluated using descriptive statistics (mean and confidence interval calculated with the standard error (SE) and 1.96 standard deviations, corrected for stratification, based on the following formula: √p(1-p)/n and with 95% confidence = p’-1.96xSE; p"+1.96SE). To analyze the differences between the scores, one-way analysis of variance (ANOVA) was applied with an alpha of 5%, and the Tukey test for means between groups was used to determine whether there were differences in the scores obtained with the FSSS, GQS, and DISCERN instruments.

To assess the agreement and reliability between observers, Fleiss's *kappa* statistic was used {K = (P_o – P_e) / (1 – P_e)}. This measure has a maximum score of 1, indicating complete agreement between evaluators, while values closer to 0 would indicate a lack of inter-observer agreement. In general, values less than 0.5 are considered unsatisfactory, while values from 0.5 to 0.75 are considered satisfactory, and values greater than 0.75 are rated as excellent.

## RESULTS

Initially, 217 *posts were identified.* Of these, 10 were excluded from the search for #frozenshoulder: 8 for not being in Portuguese and 2 for not being related to the subject. In the search for adhesive capsulitis, 7 posts were excluded: 5 because they were not in Portuguese and 2 because they were not related to the subject. Thus, 200 *posts* (100 from each topic) were evaluated using the instruments and evaluators previously mentioned.

Table 1 presents the average DISCERN, FSSS, and GQS scores, based on the scores provided by the three evaluators, with the mean and confidence interval calculated from the standard error and the tabulated standard deviation (Z = 1.96).

**Table 1:** Evaluation of mean (M), confidence interval (CI) of the analysis of variance, along with the DISCERN, FSSS, and GQS scores among 3 evaluators for shoulder and adhesive capsulitis.

Frozen shoulder	DISCERN M(CI)	FSSS M(CI)	GQS M(CI)
Shoulder and elbow surgeon	2.43 (CI= 1.70 -4.13)	2.48 (IC= 1.79 – 3.92)	2.49 (CI=2.89- 4.05)
Orthopedist	2.52* (CI= 0.95 – 4.32)	2.54 (CI =2.27 – 2.51)	2.61* (CI= 0.91 – 4.32)
General practitioner	2.42 (CI= 1.65- 3.94)	2.42 (CI =1.06- 1.24)	2.88 (CI =0.90- 4.86)
ANOVA	p > 0.05	p > 0.05	p > 0.05
**Adhesive capsulitis**	**DISCERN M(CI)**	**FSSS M(CI)**	**GQS M(CI)**
Shoulder and elbow surgeon	3.28* (CI= 1.29 -5.00)	3.26* (CI= 1.27 – 5.00)	3.50 *(CI=2.30 – 4.32)
Orthopedist	2.75 (IC= 1.35 – 4.37)	2.86 (CI=2.16 – 3.56)	2.74 (IC= 1.14 – 4.34)
General practitioner	2.40 (CI=1.35- 4.15)	2.80 (CI=1.01- 4.71)	2.87 (CI=0.97 – 4.47)
ANOVA	p > 0.05	p > 0.05	p > 0.05

* Difference between the means as seen by the Tukey test (p < 0.05).

** Difference between the means as seen by the Tukey test (p < 0.05).

*** Difference between the means as seen by the Tukey test (p < 0.05). Source: Authors.

The confidence interval was adjusted to improve precision in comparisons, using the appropriate value from the standard normal distribution for the desired confidence level (95%) and accounting for the standard error, which was estimated from the parametric nature of the data. The *post hoc* tests assessed significance in specific areas, despite the absence of statistically significant differences in the scores provided by the three evaluators.

Table 2 presents the agreement between the results indicated by the three evaluators, based on Fleiss's *kappa* statistic. It was observed that agreement among the examiners was substantial (mean k=0.75 for DISCERN, mean K=0.78 for FSSS, and mean K=0.78 for GQS), demonstrating excellent agreement (≥ 75%) among the three evaluation methodologies.

**Table 2:** Reliability and agreement regarding the scores of DISCERN, FSSS, and GQS among the three evaluators for the topics frozen shoulder and adhesive capsulitis.

Frozen shoulder	DISCERN	FSSS	GQS
Evaluators	kappa of Fleiss and 95% CI 0.81 (0.70- 0.91)	kappa of Fleiss and 95% CI 0.72 (0.65- 0.87)	kappa of Fleiss and 95% CI 0.93 (0.90- 0.96)
**Adhesive capsulitis**	**DISCERN**	**FSSS**	**GQS**
Evaluators	kappa of Fleiss and 95% CI 0.70 (0.60- 0.80)	kappa of Fleiss and 95% CI 0.84 (0.80- 0.88)	kappa of Fleiss and 95% CI 0.66 (0.60- 0.72)

Source: Authors.

## DISCUSSION

It is a fact that social networks and digital platforms provide numerous pieces of medical information. However, most of this information lacks technical and scientific validation, potentially leading to misleading guidance for patients.

Data on adhesive capsulitis/frozen shoulder available on YouTube have already been analyzed in previous studies conducted in the United States, and these studies have shown that they are of low quality and not very instructive.^10-12^ As the *posts* published on Instagram related to these topics have not yet been evaluated, this work aimed to assess the quality of this information using three objective scoring systems: the modified DISCERN, the GQS, and the FSSS. Furthermore, the interobserver reliability was evaluated, considering the results indicated by an orthopedic surgeon, a shoulder and elbow surgeon, and a general practitioner.

It is worth noting that the social network Instagram was selected as one of the most widely used in Brazil. For the study, a profile of a 50-year-old woman was created on the network, which corresponds to the sex and age most commonly affected by adhesive capsulitis/frozen shoulder.^15-17^

The analysis of the results obtained in this study demonstrates that the information available on the social network Instagram about adhesive capsulitis/frozen shoulder is, in general, of low quality. When evaluating the modified DISCERN in isolation, most posts scored between 0 and 3, indicating either a lack of information in the posts or the presence of various *fake news* and misinformation. These data also reinforce studies previously conducted on other social media platforms, such as YouTube.^10-12^

The evaluation by three professionals with varying levels of experience — an orthopedic surgeon, a shoulder and elbow specialist, and a general practitioner — with slight variation among the evaluators and high agreement, indicates that the evaluation methods used were consistent and applicable to the proposed context.^18-19^ These data align with existing literature, which demonstrates adequate interobserver reliability for validated assessment methods, such as DISCERN.^13^

The results reveal that most *posts* present incomplete information, with low technical depth and, in many cases, promotional bias. The absence of reliable references, clear explanations about symptoms, diagnosis, and therapeutic options was a constant among the analyzed content. This compromises not only the clinical utility of the publications but also the proper understanding of the disease by patients and the lay public. These findings corroborate the results of previous studies conducted on other digital platforms. Studies that evaluated YouTube videos, such as those by Tang et al.^10^, Yüce et al.^11^, and Tuna and Ay^12^, also concluded that the quality of information about adhesive capsulitis available online is limited, not very instructive, and sometimes inaccurate. Such studies reinforce the trend observed in the present work, suggesting that the problem is not exclusive to Instagram and our environment, but rather a recurring characteristic of informal medical content disseminated on social networks.

It is important to highlight that this study has significant strengths, such as the use of internationally validated scores applied by evaluators with different levels of experience, which provides methodological robustness to the analysis. Another positive aspect was the creation of a fictitious profile with demographic characteristics typical of patients with adhesive capsulitis — a 50-year-old woman — which makes the collection environment closer to reality. On the other hand, the research model adopted has inherent limitations. The dynamism of the Instagram platform, where content is frequently modified or deleted, compromises the reproducibility of the study. Furthermore, the analysis conducted in just one day represents a restricted snapshot of the universe of publications, which may not reflect variations over time.

Despite the popularity of Instagram as a source of health information, the data collected indicate that the content related to adhesive capsulitis lacks reliability, clarity, and scientific grounding. In this sense, it is essential that patients seek professional help for accurate diagnosis and treatment, avoiding reliance solely on social media for clinical decisions. It is also necessary to promote the production of high-quality educational content by health professionals to combat misinformation and foster a proper understanding of the condition.

## CONCLUSION

The quality of information available on the Instagram platform about adhesive capsulitis/frozen shoulder is unsatisfactory. The analyzed content demonstrated low reliability, a scant scientific foundation, and limited clinical utility for the lay audience, as reflected in the low scores assigned in the ratings.

## Data Availability

The contents underlying the research are available in the manuscript.
